# Rheological Characterization of Lemon Oil-Loaded Eucerin Cream and Aquaphor Ointment

**DOI:** 10.3390/ph18121838

**Published:** 2025-12-02

**Authors:** Shorouq Alzahrani, Jinsong Hao

**Affiliations:** Department of Pharmaceutical & Clinical Sciences, College of Pharmacy & Health Sciences, Campbell University, Buies Creek, NC 27506, USA

**Keywords:** rheology, lemon oil, Eucerin, Aquaphor, loading capacity

## Abstract

**Background/Objectives**: Lemon oil has demonstrated its therapeutic effects in various dermatological diseases, and different dosage forms have been investigated to incorporate lemon oil. Using the existing ointment bases to prepare drug-loaded formulations is a common practice in formulation development. Eucerin and Aquaphor are commonly used ointment bases in pharmacy for compounding preparations to meet patient needs. The maximum drug-loading capacity is an important consideration in order not to compromise desired properties of the ointment bases. The objective of this research was to determine the maximum lemon oil loading capacities by understanding the effects of loading lemon oil into Eucerin and Aquaphor on the rheological properties of the lemon oil-loaded formulations. **Methods**: Lemon oil was incorporated into Eucerin and Aquaphor at different concentrations. The rheological properties of the formulations were determined in flow and oscillation modes. Creep and recover tests were performed to determine the viscoelastic properties of the formulations. **Results**: The results showed that addition of lemon oil impacted rheological properties, including viscosity, elastic modulus, viscous modulus, compliance, and strain%, but the overall rheological behaviors remained consistent with the control for both bases. **Conclusions**: The maximum loading capacity of lemon oil could be determined from rheological testing. The rheological studies provided an important tool to determine the desired formulations for topical applications, without affecting the performance characteristics of the commercial products.

## 1. Introduction

Derived from cold pressing the outer segments of the lemon pericarp, lemon oil is a vital essential oil with a potent lemon fragrance. It plays a significant role in the pharmaceutical industry as a primary botanical source. It is included in the monographs of the United States, European, and Indian Ayurvedic Pharmacopoeias. The array of compounds found in lemon oil, such as flavonoids, carboxylic acids, coumarins, phenolic acids, vitamins, amino acids, and essential monoterpenoids, contribute to its potential applications in the pharmaceutical, cosmetic, and culinary industries [1]. Lemon oil has antioxidative [2], antibacterial [3], antifungal [4], and anticancer [5] properties. It has been widely used in aromatherapy and aroma massage [6,7], and recently studied for wound healing [8]. It is also a potential penetration enhancer to increase topical and transdermal drug delivery [9,10]. Various dosage forms have been investigated to incorporate lemon oil, including nanoemulsions [11], nanoparticles [12], micelles [13], emulgels [14], niosomes [15], and cyclodextrin complexes [16]. Incorporating an active pharmaceutical ingredient (API) into an existing semisolid product is a widely used technique in compounding pharmacy for creating customized medications to meet unique patient needs. This strategy also has practical applications in pharmaceutical formulation development.

Aquaphor is a safe and effective ointment-based formulation and a top emollient on the market. Eucerin, a cream-based product, is well-known for its favorable pharmacological attributes, including preventing and treating dry skin and minor skin irritation. According to the United States Pharmacopeia (USP), both Aquaphor and Eucerin are classified as absorption bases and are useful as emollients. These absorption bases contain an oleaginous material and a water-in-oil emulsifier, so that they can either contain water in the formulation to form a w/o emulsion base (i.e., emulsion absorption bases) or form a w/o emulsion in situ after absorbing water (i.e., anhydrous absorption bases). Eucerin is a w/o emulsion of petrolatum, mineral oil, mineral wax, wool wax, alcohol, and bronopol. Aquaphor is an anhydrous absorption ointment base, containing petrolatum, mineral oil, mineral wax, and wool wax alcohol. Eucerin and Aquaphor are commonly used ointment bases in dermatological formulations [17,18]. Aquaphor demonstrated its wound healing effect in rats [19]. Many drugs were incorporated into Eucerin for wound healing studies [20,21,22]. Eucerin-based ointments containing lemon oil of 33% *w*/*w* enhanced healing of infected wounds in rats [8]. Aquaphor and Eucerin are the top choice ointments for superficial burns [23]. Therefore, they were chosen to load lemon oil in this study.

Retaining the desired properties of bases like Eucerin and Aquaphor is important, particularly when incorporating a large amount of oil, because oil can potentially weaken or disrupt the base structure, decrease the volume fraction of the internal phase, dilute the continuous phase, and lower the concentration of emulsifiers, leading to stability changes [24,25,26]. Careful considerations of loading capacity and formulation composition are thus needed. Rheological testing can help determine loading capacity and rheological properties of the ointment bases, which are complex multiphase systems. Rheological behaviors can be evaluated by flow rheology, oscillatory rheology, and creep testing. In flow rheology, flow and viscosity curves are obtained as functions of shear rate. In oscillatory rheology, the test is performed in a linear viscoelastic region, where the microstructure of formulation is not destructed, so that the elastic component and viscous component can be investigated. Creep and creep/recovery tests are other methods to determine the rheological properties when the creep test is performed in a linear viscoelastic region. These rheological testing methods have been investigated in various aspects of semisolid formulations, such as formulation development, quality control, and bioequivalence evaluation. For example, rheological testing was used in understanding drug–polymer and polymer–polymer interactions in gel formulations [27,28], petrolatum structures [29,30], and generic product equivalence of topical formulations [31,32].

Rheological testing was used in this research to determine the maximum capacities of lemon oil that Eucerin and Aquaphor can hold without affecting their desired rheological characteristics. Lemon oil was loaded to Eucerin and Aquaphor ointment bases at different concentrations. The rheological properties of the formulations were measured in flow and oscillation modes. A creep test was also performed to determine the viscoelastic properties of the formulations. The effects of incorporating lemon oil into Eucerin and Aquaphor on the rheological properties of lemon oil-loaded formulations were investigated. The results of this study will not only provide valuable insights into the rheological behaviors of lemon oil–Eucerin cream and lemon oil–Aquaphor ointment formulations, but also help determine the most suitable concentrations of lemon oil in these bases for achieving optimal skincare performance, taking advantage of lemon oil’s numerous benefits and the distinct characteristics of Eucerin and Aquaphor.

## 2. Results

### 2.1. Flow Measurements

**Figure 1:**
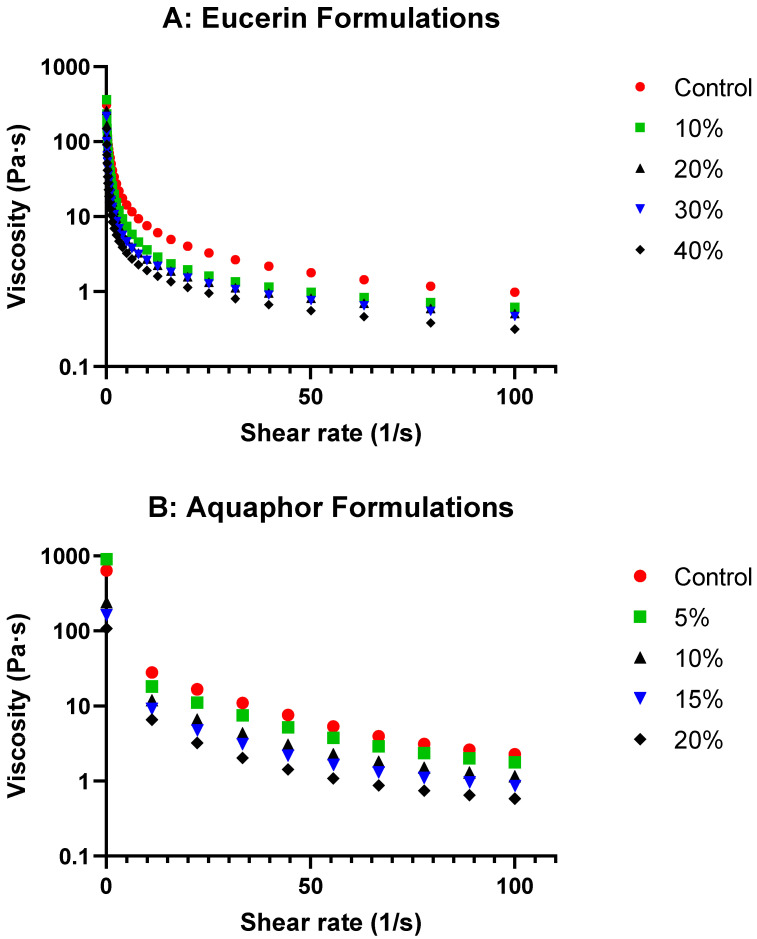
Viscosity profiles of lemon oil-loaded (**A**) Eucerin and (**B**) Aquaphor formulations as functions of shear rate.

Figure 1 presents the viscosity curves for Eucerin and Aquaphor formulations and Table S1 in the Supplementary Section summarizes the viscosity values at representative shear rates. Viscosity decreased with increasing shear rate for both formulation series, indicating pseudoplastic flow behaviors. However, the addition of lemon oil did not change the shear-thinning property at the concentrations of lemon oil investigated. With increasing temperatures, the viscosity decreased for both Aquaphor control and Aquaphor 15% formulations, but the changes in the viscosity were slightly less for the Aquaphor 15% than for the Aquaphor control (see Figure S4). For the Aquaphor control, the viscosities changed by 72% (from 166 Pa·s at 25 °C to 46 Pa·s at 37 °C). The viscosities of Aquaphor 15% changed by 60% (from 55 Pa·s at 25 °C to 22 Pa·s at 37 °C).

### 2.2. Oscillation Measurements

**Figure 2:**
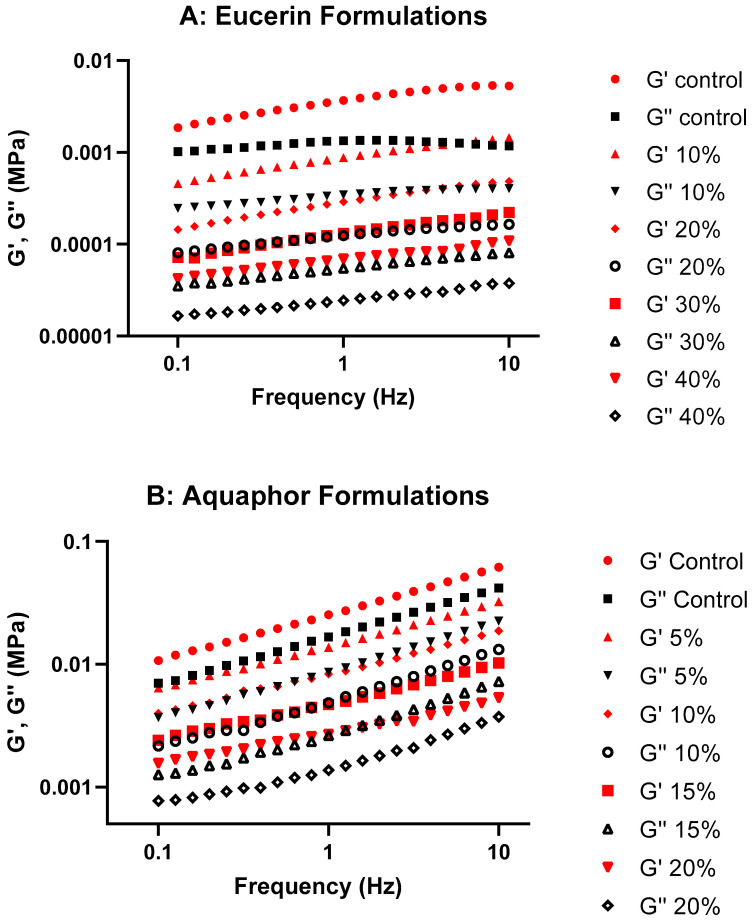
Plots of storage modulus (G′) and loss modulus (G″) over oscillatory frequency for lemon oil-loaded (**A**) Eucerin and (**B**) Aquaphor formulations.

**Figure 3:**
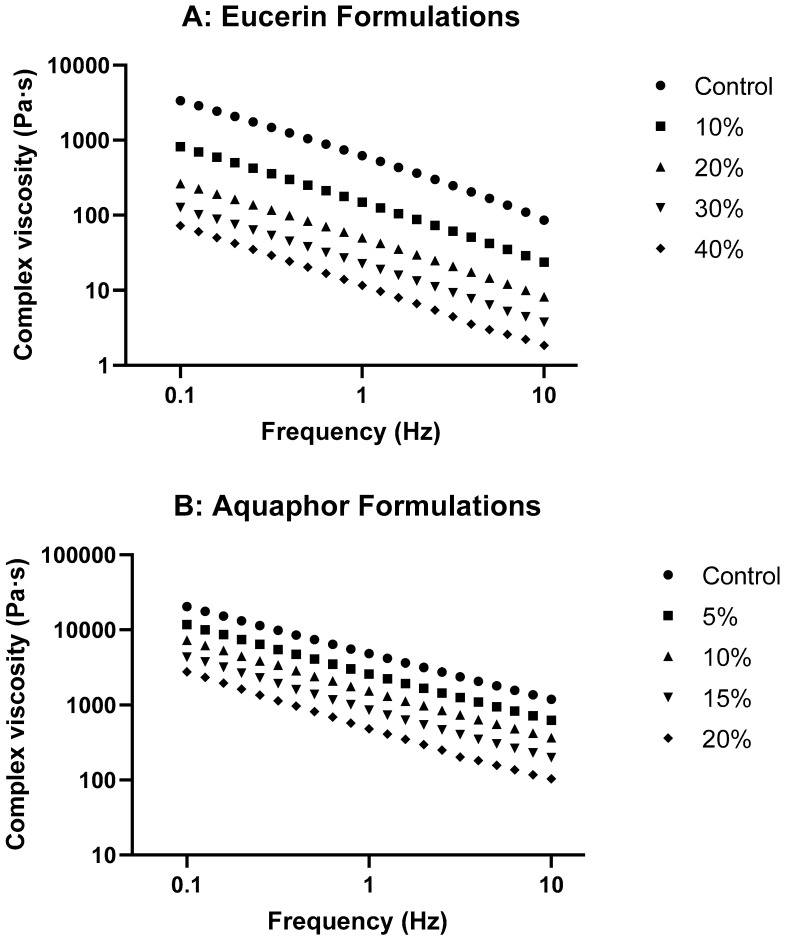
Complex viscosity as a function of oscillatory frequency for lemon oil-loaded (**A**) Eucerin and (**B**) Aquaphor formulations.

Figure 2 presents the changes in G′ and G″ over oscillatory frequency for both formulation series. Both G′ and G″ were generally increased with frequency for formulations. G′ values were higher than G″ for each formulation. No crossover of G′ and G″ occurred in the frequency range measured. These suggested frequency-dependent viscoelastic properties with greater elastic than viscous components for all formulations. When the concentration of lemon oil in the formulations increased, both the G′ and G″ decreased, indicating the effects of lemon oil on the viscoelastic properties. The effect of lemon oil on complex viscosity in Eucerin and Aquaphor formulations is shown in Figure 3. Complex viscosity decreased with oscillation frequency; increasing the concentration of lemon oil decreased the viscosity at the same oscillation frequency. There were no time-dependent changes in G′ and G″ for either Aquaphor control or Aquaphor 15% formulations, suggesting stable viscoelastic behaviors of the formulations (see Figure S5).

### 2.3. Creep Testing

**Figure 4:**
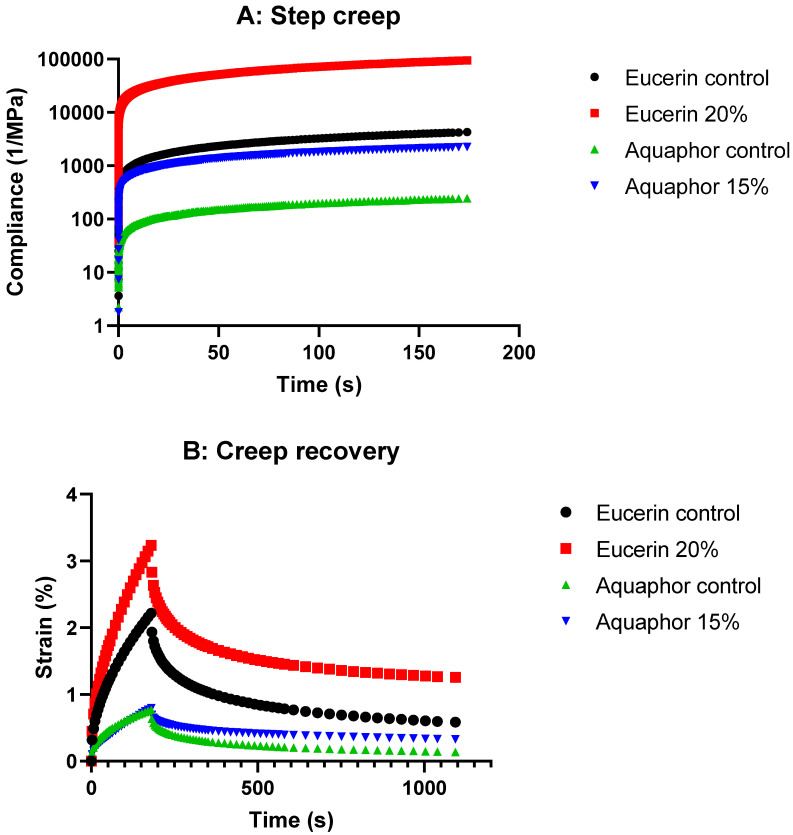
The effects of lemon oil on (**A**) step creep and (**B**) recovery of Eucerin and Aquaphor formulations with and without lemon oil.

Figure 4A presents deformation of the formulations under representative stress in a linear viscoelastic region. The compliance values for the lemon oil-loaded formulations were greater than the corresponding controls, suggesting easier deformation. Upon the removal of the stress applied in the recovery phase (Figure 4B), the lemon oil-loaded formulations had greater strain% than their controls, respectively. These results indicated that the incorporation of lemon oil made the formulations more mobile. Both Eucerin and Aquaphor formulations followed the same patterns; however, the deformation was easier for the Eucerin formulations than the Aquaphor formulations, as evidenced by the higher compliance (Figure 4A) or strain% (Figure 4B) values.

On closer observation of Figure 4A, it becomes apparent that the creep compliance–time curve for each formulation was composed of three regions, reflecting an instantaneous elastic deformation, a delayed elastic deformation, and a viscous deformation, respectively. The lemon oil-loaded formulations showed greater initial compliance immediately after the stress was applied and greater maximum compliance before the stress was removed, as compared with their respective control formulations. These indicated the formulations were viscoelastic, and the addition of lemon oil changed the contribution of elastic and viscous components.

Similarly, the recovery curve for each formulation in the recovery phase (Figure 4B) also consisted of three regions. The strain decreased immediately upon the removal of the stress, then decreased gradually, and finally decreased linearly with time. These strain changes suggested that the recovery started from the instantaneous elastic deformation, followed by the delayed elastic deformation, and the strain did not recover to the original value due to the viscous flow. The lemon oil-loaded formulations had higher strain%, indicating more viscous deformation contribution than their respective control formulations.

## 3. Discussion

Determining the maximum loading capacities of lemon oil in Eucerin and Aquaphor was a crucial aspect of this study. Through a meticulous process of gradually incorporating lemon oil into the bases, careful observation of the rheological properties was undertaken to ensure that the structure and consistency of the formulations were not compromised. It was found that Eucerin could accommodate up to approximately 50% lemon oil before any signs of instability or undesirable rheological behavior were observed, highlighting its considerable loading capacity for lemon oil. On the other hand, Aquaphor exhibited a lower maximum loading capacity, reaching its threshold at around 25% lemon oil concentration. This difference in loading capacities suggests that the inherent properties and formulation of Eucerin may allow it to incorporate better and maintain the stability of higher concentrations of lemon oil. At the same time, Aquaphor may have a more limited ability to integrate the oil without affecting its overall structure and performance. To verify the instabilities or rheological behavior changes, oscillation frequency sweep tests were performed in our preliminary studies. Figure S1 shows the viscoelastic behavior changes when the concentration of lemon oil reached its maximum. For Eucerin formulations, G′ curve was above G″ curve for both Eucerin control and Eucerin 50%, but the two curves tended to be parallel for Eucerin 50% and cross at frequencies below 0.1 Hz for Eucerin control. In contrast, Aquaphor formulations showed different patterns. G′ curve was above G″ curve for both Aquaphor control and Aquaphor 20%, but the two curves tended to be parallel for the control and cross at frequencies above 10 Hz for Aquaphor 20%. Therefore, the maximum concentrations of lemon oil were set as 40% for Eucerin and 20% for Aquaphor. The oscillation frequency was in the range of 0.1 to 10 Hz. The determination of these maximum loading capacities is essential for optimizing the formulations, ensuring that the benefits of lemon oil can be harnessed without compromising the desired rheological properties of the Eucerin and Aquaphor bases.

The rheological study of the formulations with varying lemon oil concentrations in Eucerin and Aquaphor revealed exciting insights into their behaviors. The viscosity curves (Figure 1 and Figure 3) showed decreases in viscosity as the lemon oil concentration increased while maintaining the same flow trend for both bases. This suggests that, although the formulations become less viscous, flow behavior remains consistent with the control, indicating that adding lemon oil does not significantly alter the overall flow characteristics of the bases. As expected, the formulations became less viscous at higher temperatures (Figure S4). The temperature-dependent changes in viscosity were less drastic with the addition of lemon oil, likely due to the reduced relative portion of solid components in the formulations. These attributes are desired for topical application.

Storage modulus is proportional to the extent of the elastic component, which is usually contributed by crosslinking, entanglement, and/or aggregation of the system. Loss modulus is proportional to the extent of the viscous component, which is usually contributed by the liquid-like portion. As there are significant internal structures in ointments and creams, they cannot relax quickly and are highly elastic; G′ is expected to be higher than G″ at the same frequency. The Eucerin and Aquaphor formulations in this research showed higher G′ than G″ at the frequency range studied without crossover, suggesting the dominance of the elastic properties and the stability of the structures. In the G′ and G″ graphs (Figure 2), the storage and loss moduli exhibit the same trend as the viscosity curves, with a decrease in values as the lemon oil concentration increased for both Eucerin and Aquaphor. This observation implies that the elastic and viscous properties of the formulations were affected by the addition of lemon oil. Nevertheless, overall viscoelastic behavior remained consistent with the control for both bases.

The step creep and creep/recovery graphs (Figure 4) show higher compliance values with adding lemon oil while still following the same trend as the control for both Eucerin and Aquaphor. This finding suggests that the formulations became slightly more susceptible to deformation under constant applied stress with the addition of lemon oil. However, their overall rheological behavior remained consistent with the control, maintaining mechanical strength and stability for both bases.

Viscoelastic materials exhibit a nonlinear response to strain. Both Eucerin and Aquaphor formulations were viscoelastic; each creep or recovery curve can be subdivided into three regions, following the typical creep or recovery behaviors of a viscoelastic material. The value and the shape of the creep and recovery curves are important for understanding the effects of addition of lemon oil. Upon the application of stress, the bonds between the primary structural units of the formulations stretched elastically in the region of instantaneous compliance, resulting in sudden increase in compliance. When the stress was continuously applied, some bonds ruptured in the time-dependent retarded elastic region, leading to nonlinear increase in the compliance with time. The ruptured bonds then allowed the flow of the formulations in the viscous region, where the compliance increased linearly with time and the steady-state compliance was obtained. Upon removal of the stress, first, there was an instantaneous elastic recovery, which corresponded to the instantaneous elastic deformation in the creep curve. The retarded elastic recovery region was equivalent to the retarded elastic region of the creep curve. As some bonds were irreversibly broken in the creep region, the original structure was not recovered completely. The final deformation was less than the initial deformation due to the ability of viscoelastic materials to partially recover structure by storing energy. As the addition of lemon oil made the formulation deform easily, the compliance and strain% values were greater than the control formulations. However, the viscoelastic behaviors were not changed, implying the same applications of the lemon oil-loaded formulations for their intended uses as commercial Eucerin and Aquaphor products. The results of creep recovery tests supported the results of flow and oscillation testing.

While both Eucerin and Aquaphor are absorption bases containing petrolatum and w/o surfactants, Aquaphor is an anhydrous base and Eucerin is a w/o emulsion base. Aquaphor contains no water, but high solid content (> 45% petrolatum). Eucerin contains water in the internal phase of the emulsion base, but low petrolatum content. Petrolatum is considered the major contributor to the rheological properties of finished ointment products [33]. Petrolatum can form an intertwined network of microcrystalline lamellar sheets in semisolid formulations [29]. Studies on petrolatum rheology [34,35] have shown that petrolatum alone shows a shear-thinning flow and viscoelasticity with elastic dominance. The addition of paraffin oil to petrolatum weakens the structure, and mixtures become less shear-dependent [24]. This weakening is attributed to the increase in the liquid phase within the mixture. When the increase in the liquid phase saturates the voids in the structure, the crystalline structure breaks down, resulting in a loss of strength [24]. The absorption bases in the present research also showed viscoelastic properties. They may be pictured as a dispersion of molecules with intermittent spring-type segments in a highly viscous mass. Petrolatum molecules interact with each other and behave as springs, contributing to elasticity. The continuous viscous mass can resemble a dashpot, contributing to viscosity. The deformation of the springs is retarded by the surrounding viscous mass. Aquaphor can have a stronger network formation than Eucerin due to the greater fraction of petrolatum in the formulation. Adding lemon oil to Aquaphor can weaken the interactions between petrolatum molecules and make the continuous mass thinner. Disrupting the solid network structure of petrolatum will reduce its resistance to flow, with the effect becoming more pronounced as the oil content increases. Eucerin is a water-in-oil emulsion base. The internal water droplets disperse in the continuous petrolatum network. Increased volume fraction of the internal phase and more pronounced crystal network in the continuous phase are expected to increase viscosity and storage modulus and decrease compliance [25,26]. The addition of lemon oil to Eucerin can decrease the volume fraction of the internal phase and weaken the interactions of petrolatum molecules in the continuous phase, causing more widely dispersed internal droplets in less viscous continuous mass.

The differences in the type and structure of the two bases can be reflected in the differences in the rheological properties of the formulations. For example, Aquaphor control showed higher viscosity and G′ and G″, and lower compliance and strain%, as compared to Eucerin control. These suggested greater structure in the Aquaphor ointment than in the Eucerin cream, which was associated with the nature of ointment bases. Aquaphor was more solid-like than Eucerin. Oscillation frequency sweep tests revealed different viscoelastic performances between Aquaphor and Eucerin (Figures S1–S3 and Table S2). Both Aquaphor and Eucerin controls had tan δ values less than 1 over the frequencies measured. Tan δ values trended down with frequency for Eucerin control (with a negative slope) but trended up with frequency for Aquaphor control (with a positive slope). G′ and G″ curves of Eucerin control tended to cross over at frequencies below 0.1 Hz, but the curves of Aquaphor were parallel without crossover.

With the incorporation of lemon oil, the relative contribution of elastic and viscous components to the overall viscoelastic properties were changed. When the concentration of lemon oil was increased, the plots of tan delta versus frequency tended to level off for Eucerin formulations and trend up for Aquaphor formulations (Figures S2 and S3). The slope of the tan delta versus frequency curve was linearly increased with lemon oil concentration for both Eucerin and Aquaphor formulations (Table S2), despite negative slopes for Eucerin formulations and positive slopes for Aquaphor formulations. With increasing lemon oil concentration, G′ and G″ curves of Eucerin formulations became more parallel to each other, but the curves of Aquaphor formulations tended to cross over at high frequencies. Increasing concentration of lemon oil increased the viscous contribution; Aquaphor formulations behaved more like viscoelastic fluids, but Eucerin formulations tended to have balanced elastic and viscous responses at all frequencies measured. Adding lemon oil to Eucerin diluted the emulsion base, which decreased the volume fraction of the disperse phase and the concentrations of surfactants, leading to decreased consistency, smaller G′ and G″, more viscous contribution, and easier deformation. Lemon oil could act as a lubricant in the Aquaphor formulations, weakening the structure of Aquaphor. The lemon oil-loaded Aquaphor formulations thus showed lower viscosity, smaller G′ and G″, higher compliance, and higher strain% than Aquaphor control. Eucerin emulsion base absorbed more lemon oil, but Aquaphor had limited capability to accommodate lemon oil without softening the semisolid. Therefore, the addition of lemon oil had smaller effects on Aquaphor than on Eucerin.

Rheological testing has been widely used to determine the desired properties of semisolid formulations for applications. Rotational experiments provide information on the flow properties of a system; dynamic oscillation testing reveals the microscopic structure of a viscoelastic material; and creep/recovery testing is an alternative for obtaining the relaxation time and viscoelastic properties of a material. In this research, three different rheological testing methods were used, and the formulations were tested under conditions where the microstructure of the formulations was either destructed or not destructed. Creep testing was also performed to complement rotation testing. The results from these different tests were found consistent, suggesting that the lemon oil made Eucerin and Aquaphor ointment bases less elastic and more mobile; therefore, the formulations would be easier to apply. These tests complemented each other and provided better understanding of the effects of lemon oil on the rheological behaviors of the formulations. They also allowed for determination of the desired concentrations of lemon oil and its loading capacity. Levigation of a solid drug with a liquid levigating agent or incorporation of a liquid to an existing commercial semisolid product is a common practice in compounding pharmacy. The present study further supports that rheological testing can be an important tool to determine the loading capacity of semisolids.

## 4. Materials and Methods

### 4.1. Materials

Eucerin Advanced Repair Cream (Beiersdorf Inc., Stamford, CT, USA), Aquaphor Healing Ointment (Beiersdorf Inc., Wilton, CT, USA), and lemon oil (expressed, cold pressed, California type, Spectrum Chemical, Gardena, CA, USA) were used as received.

### 4.2. Determination of Maximum Loading Capacity

To ascertain the upper limit of lemon oil incorporation in the base cream or ointment without adversely affecting the rheological properties of the formulation, stepwise addition of lemon oil to the Eucerin base cream or Aquaphor ointment base was employed. The procedure commenced with introducing a minimal quantity of lemon oil, followed by manual blending utilizing a spatula. Continuous monitoring of the mixture was conducted to identify any changes in consistency and texture. The steps of adding lemon oil gradually and achieving uniformity were repeated. It was found that the formulations became less stable when the concentrations of lemon oil were increased to 50% *w*/*w* for Eucerin series and 25% *w*/*w* for Aquaphor series. Instabilities were verified by oscillation frequency sweep tests in our preliminary studies, and the results are presented in Figure S1 in the Supplementary Section. Therefore, the maximum concentrations of lemon oil used in the following testing were 40% *w*/*w* for Eucerin formulations and 20% *w*/*w* for Aquaphor formulations.

### 4.3. Formulation of Lemon Oil–Eucerin Cream and Lemon Oil–Aquaphor Ointment

Different concentrations of lemon oil were added to formulate lemon oil–Eucerin creams and lemon oil–Aquaphor ointments. Eucerin-based creams contained 10%, 20%, 30%, and 40% *w*/*w* lemon oil (i.e., Eucerin 10%, 20%, 30%, and 40%). Aquaphor ointment samples contained 5%, 10%, 15%, and 20% *w*/*w* lemon oil (i.e., Aquaphor 5%, 10%, 15%, and 20%). Lemon oil was gradually incorporated into Eucerin base cream or Aquaphor ointment base through manual mixing with a spatula following a standardized procedure. Upon achieving a uniform consistency, the prepared samples were transferred to airtight containers and stored under controlled conditions (at 25 °C) until further analysis. The varying concentrations of lemon oil in both the Eucerin cream and Aquaphor ointment formulations allowed for comprehensive evaluation of their rheological behaviors. Eucerin and Aquaphor without lemon oil were used as controls (Eucerin control and Aquaphor control) in the rheological testing.

### 4.4. Rheological Measurements

Rheological testing of lemon oil-loaded Eucerin cream and Aquaphor ointment samples was performed using a TA Instruments Discovery Hybrid Rheometer-2 (DHR-2, New Castle, DE, USA) equipped with either a 40 mm sandblasted or a 20 mm smooth parallel plate sensor system. A 1000 µm gap was set for the measurement, and a 1050 µm trimming gap was used to ensure uniform sample thickness. To mimic skin conditions during testing, the rheometer’s temperature control system was set to maintain a constant temperature of 32 °C, representing the average human skin temperature. The rheological measurements were performed at 32 °C, except for flow temperature ramp tests. Before initiating the rheological measurements, a 5 min equilibration time was allowed for the samples to adjust to the set temperature, ensuring consistent and accurate results. The measurements were performed in both flow and oscillation modes. The series of Eucerin and Aquaphor formulations containing different concentrations of lemon oil were used in tests including flow continuous ramp, oscillation amplitude sweep, and oscillation frequency sweep. Eucerin-based formulations were Eucerin control, Eucerin 10%, Eucerin 20%, Eucerin 30%, and Eucerin 40%. Aquaphor-based formulations were Aquaphor control, Aquaphor 5%, Aquaphor 10%, Aquaphor 15%, and Aquaphor 20%. For other tests described below, only representative formulations (Eucerin control, Eucerin 20%, Aquaphor control, and Aquaphor 15%) were selected for evaluation.

#### 4.4.1. Flow Continuous Ramp

The flow curves of the preparations were determined in flow ramp tests. The shear rates used ranged from 1 to 100 s^−1^. The formulations tested included Eucerin-based formulations (Eucerin control, Eucerin 10%, Eucerin 20%, Eucerin 30%, and Eucerin 40%) and Aquaphor-based formulations (Aquaphor control, Aquaphor 5%, Aquaphor 10%, Aquaphor 15%, and Aquaphor 20%). Viscosity profiles of lemon oil-loaded Eucerin and Aquaphor formulations as functions of shear rate are presented in Figure 1. Viscosity values at representative shear rates for both Eucerin and Aquaphor formulations are summarized in Table S1 in the Supplementary Section.

#### 4.4.2. Oscillation Amplitude and Frequency Sweep

Oscillation amplitude sweep tests were first performed from 0.1 to 100 Pa at an oscillatory frequency of 1 Hz to determine the linear viscoelastic region for each formulation. Eucerin formulations included Eucerin control, Eucerin 10%, Eucerin 20%, Eucerin 30%, and Eucerin 40%; Aquaphor formulations included Aquaphor control, Aquaphor 5%, Aquaphor 10%, Aquaphor 15%, and Aquaphor 20%. The linear viscoelastic ranges for Eucerin and Aquaphor formulations were 0.5–10 Pa and 2–15 Pa, respectively.

A stress value representative of the linear viscoelastic region was chosen for the frequency sweep test. The oscillatory measurements were performed over a frequency range of 0.1 to 10 Hz at the selected stress. The rheological parameters, including storage modulus or elastic modulus (G′), loss modulus or viscous modulus (G″), complex viscosity (η*), and tan delta (tan δ) were obtained. G′ is a measure of the energy stored and recovered per cycle of deformation and reflects the solid-like component of a viscoelastic material. G″ is a measure of the energy lost per cycle and reflects the liquid-like component. η* is a measure of the total resistance to flow. Tan δ, which is defined as the ratio of the viscous modulus to the elastic modulus, is an indicator of the overall viscoelasticity of the sample, decreasing with increased elasticity of the test system. Plots of G′ and G″ over oscillatory frequency for lemon oil-loaded Eucerin and Aquaphor formulations are presented in Figure 2, as well as in Figure S2 (for Eucerin formulations) and Figure S3 (for Aquaphor formulations) in the Supplementary Section. Changes in complex viscosity as functions of oscillatory frequency for lemon oil-loaded Eucerin and Aquaphor formulations are presented in Figure 3. Plots of tan δ versus oscillatory frequency are presented in Figure S2 (for Eucerin formulations) and Figure S3 (for Aquaphor formulations) in the Supplementary Section. The slopes of the tan δ curves and their linear relationships with lemon oil concentration are summarized in Table S2 in the Supplementary Section.

#### 4.4.3. Step Creep and Recovery

Creep measurements were conducted in step creep mode for Eucerin control, Eucerin 20%, Aquaphor control, and Aquaphor 15% formulations. The stress representative of the linear viscoelastic region was applied, i.e., 5 Pa for Eucerin control, 0.5 Pa for Eucerin 20%, 15 Pa for Aquaphor control, and 2 Pa for Aquaphor 15%. The compliance values were obtained as functions of time (Figure 4).

To determine the recovery behaviors of the formulations, they were subjected to creep and recovery testing. During the creep phase, a stress representative of the linear viscoelastic region was applied (i.e., 5 Pa for Eucerin control, 0.5 Pa for Eucerin 20%, 15 Pa for Aquaphor control, and 2 Pa for Aquaphor 15%). During the recovery phase, the stress was removed and the strain% was determined as a function of time. Effects of lemon oil on creep and recovery of Eucerin and Aquaphor formulations are presented in Figure 4.

#### 4.4.4. Flow Temperature Ramp

To determine the effects of temperature on rheological properties of the formulations, Aquaphor control and Aquaphor 15% formulations were tested in a flow temperature ramp mode at a shear rate of 1 s^−1^. The temperature increased from 25 °C to 37 °C at a ramp rate of 5 °C/min. Viscosity profiles of Aquaphor control and Aquaphor 15% as functions of testing temperature are presented in Figure S4 in the Supplementary Section.

#### 4.4.5. Oscillation Time Sweep

Oscillation time sweep tests were performed with Aquaphor control and Aquaphor 15% formulations in a linear viscoelastic region with a representative stress applied. The oscillatory frequency was set at 1 Hz. Plots of G′ and G″ over time for Aquaphor control and Aquaphor 15% formulations are presented in Figure S5 in the Supplementary Section.

#### 4.4.6. Reproducibility Testing

To demonstrate testing reproducibility, Eucerin 20% and Aquaphor 15% were evaluated in triplicate using oscillation frequency sweep under the same conditions as described in the oscillation frequency sweep section. Plots of G′ and G″ over oscillatory frequency for Eucerin 20% and Aquaphor 15% formulations are presented in Figure S6 in the Supplementary Section. The results suggested the reproducibility of the testing; tests were, thus, typically performed in replicates of 2–3.

## 5. Conclusions

The rheological study of the Eucerin and Aquaphor formulations with varying lemon oil concentrations revealed that, while adding lemon oil impacts some properties, such as viscosity, G′, G″, and compliance, overall rheological behavior remains consistent with the control for both bases. This consistency suggests that the formulations may still be suitable for their intended applications, providing desirable performance characteristics, such as spreadability, skin penetration, and drug release, while maintaining mechanical strength and stability. Rheological testing could help determine the maximum lemon oil loading capacities to the existing ointment bases.

## Data Availability

The original data has been presented in the article. Further inquiries can be directed to the corresponding author.

## Supplementary Materials

The following supporting information can be downloaded at: https://www.mdpi.com/article/10.3390/ph18121838/s1, Table S1: Viscosities (Pa·s) at representative flow rates (s−1) for Eucerin-based and Aquaphor-based formulations containing various concentrations (% *w*/*w*) of lemon oil measured in flow sweep tests. Table S2: Slopes of the plots of tan delta versus frequency for Eucerin and Aquaphor formulations containing various concentrations (% *w*/*w*) of lemon oil measured in flow sweep tests. The slopes were linearly changed with lemon oil concentration. Figure S1: Plots of storage modulus (G′), loss modulus (G″), and tan delta (tan δ) over oscillatory frequency obtained in the preliminary oscillation frequency sweep studies to determine maximum loading of lemon oil for Eucerin and Aquaphor formulations. Figure S2: Plots of storage modulus (G′), loss modulus (G″), and tan delta (tan δ) over oscillatory frequency for lemon oil-loaded Eucerin formulations. Figure S3: Plots of storage modulus (G′), loss modulus (G″), and tan delta (tan δ) over oscillatory frequency for lemon oil-loaded Aquaphor formulations. Figure S4: Viscosity profiles of Aquaphor control and Aquaphor 15% formulations as functions of testing temperature. Figure S5: Plots of storage modulus (G′) and loss modulus (G″) over time for Aquaphor control and Aquaphor 15% formulations. Figure S6: Plots of storage modulus (G′) and loss modulus (G″) over oscillatory frequency for (A) Eucerin 20% and (B) Aquaphor 15% formulations after three tests under the same testing conditions.

## References

[1] Klimek-Szczykutowicz M., Szopa A., Ekiert H. (2020). *Citrus limon* (lemon) phenomenon-a review of the chemistry, pharmacological properties, applications in the modern pharmaceutical, food, and cosmetics industries, and biotechnological studies. Plants.

[2] Calabrese V., Scapagnini G., Randazzo S.D., Randazzo G., Catalano C., Geraci G., Morganti P. (1999). Oxidative stress and antioxidants at skin biosurface: A novel antioxidant from lemon oil capable of inhibiting oxidative damage to the skin. Drugs Exp. Clin. Res..

[3] Luciardi M.C., Blázquez M.A., Alberto M.R., Cartagena E., Arena M.E. (2021). Lemon oils attenuate the pathogenicity of *Pseudomonas aeruginosa* by quorum sensing inhibition. Molecules.

[4] Białoń M., Krzyśko-Łupicka T., Koszałkowska M., Wieczorek P.P. (2014). The influence of chemical composition of commercial lemon essential oils on the growth of *Candida* strains. Mycopathologia.

[5] Manjunath C., Mahurkar N. (2021). In vitro cytotoxicity of cardamom oil, lemon oil, and jasmine oil on human skin, gastric, and brain cancer cell line. J. Cancer Res. Ther..

[6] Döner Ş., Dağ Tüzmen H., Duran B., Sunar F. (2024). The effect of aromatherapy massage with lemon and peppermint essential oil on menopausal symptoms: A double-blinded, randomized placebo controlled clinical trial. Explore.

[7] Mori M., Ikeda N., Kato Y., Minamino M., Watabe K. (2002). Inhibition of elastase activity by essential oils in vitro. J. Cosmet. Dermatol..

[8] Valizadeh A., Shirzad M., Pourmand M.R., Farahmandfar M., Sereshti H., Amani A. (2019). Preparation and comparison of effects of different herbal oil ointments as wound-healing agents. Cells Tissues Organs.

[9] Subramaniyan G., Rubina S., Ramana B.V., Stanley A.M., Srinivasan D. (2024). Aloe vera leaf mucilage and lemon oil as potential penetration-enhancing agents to increase lornoxicam transdermal administration using nano vesicular gel. Pak. J. Pharm. Sci..

[10] Valgimigli L., Gabbanini S., Berlini E., Lucchi E., Beltramini C., Bertarelli Y.L. (2012). Lemon (*Citrus limon*, Burm.f.) essential oil enhances the trans-epidermal release of lipid-(A, E) and water-(B_6_, C) soluble vitamins from topical emulsions in reconstructed human epidermis. Int. J. Cosmet. Sci..

[11] Azmi N.A.N., Elgharbawy A.A.M., Salleh H.M., Moniruzzaman M. (2022). Preparation, characterization and biological activities of an oil-in-water nanoemulsion from fish by-products and lemon oil by ultrasonication method. Molecules.

[12] Gholamhossein Tabar Valookolaei F.S., Sazegar H., Rouhi L. (2025). The antibacterial capabilities of alginate encapsulated lemon essential oil nanocapsules against multi-drug-resistant *Acinetobacter baumannii*. Sci. Rep..

[13] Man A., Santacroce L., Jacob R., Mare A., Man L. (2019). Antimicrobial activity of six essential oils against a group of human pathogens: A comparative study. Pathogens.

[14] Tello P., Calero N., Santos J., Trujillo-Cayado L.A. (2023). Development of avocado and lemon oil emulgels based on natural products: Phycocyanin and pectin. Pharmaceutics.

[15] Eid R.K., Essa E.A., El Maghraby G.M. (2019). Essential oils in niosomes for enhanced transdermal delivery of felodipine. Pharm. Dev. Technol..

[16] Bhandari B.R., D’Arcy B.R., Padukka I. (1999). Encapsulation of lemon oil by paste method using β-cyclodextrin: Encapsulation efficiency and profile of oil volatiles. J. Agric. Food Chem..

[17] Anwar Y., Lowenstein E.J. (2016). Eucerin: A revolutionary formulation still going strong for over a century. Skinmed.

[18] Metry D.W., Hebert A.A. (2000). Topical therapies and medications in the pediatric patient. Pediatr. Clin. N. Am..

[19] Little J.W., Bickley H.C., Daugherty W.B., Bickley C. (1972). Effect of Aquaphor ointment on wound healing. J. Dent. Res..

[20] Fallah Huseini H., Abdolghaffari A.H., Ahwazi M., Jasemi E., Yaghoobi M., Ziaee M. (2020). Topical application of teucrium polium can improve wound healing in diabetic rats. Int. J. Low. Extrem. Wounds.

[21] Nasiry D., Khalatbary A.R., Ghaemi A., Ebrahimzadeh M.A., Hosseinzadeh M.H. (2022). Topical administration of *Juglans regia* L. leaf extract accelerates diabetic wound healing. BMC Complement. Med. Ther..

[22] Bobiński R., Wyszomirski M., Machnickam A., Pielesz A., Kawecki M., Waksmańska W., Staniszewski L. (2020). The effect of lauric acid on pathogens colonizing the burn wound: A pilot study. Altern. Ther. Health Med..

[23] Liu H.Y., Alessandri Bonetti M., Shockey S., Elias G.A., Corcos A.C., Ziembicki J.A., Egro F.M. (2025). Cross-sectional study on the management of nonoperative burns at American Burn Association-verified burn centers. J. Burn. Care Res..

[24] Duarte J.C., Schellart W.P., Cruden A.R. (2014). Rheology of petrolatum–paraffin oil mixtures: Applications to analogue modelling of geological processes. J. Struct. Geol..

[25] Haj-shafiei S., Ghosh S., Rousseau D. (2013). Kinetic stability and rheology of wax-stabilized water-in-oil emulsions at different water cuts. J. Colloid. Interface Sci..

[26] Rafanan R., Rousseau D. (2017). Dispersed droplets as active fillers in fat-crystal network-stabilized water-in-oil emulsions. Food Res. Int..

[27] Badruddoza A.Z.M., Yeoh T., Shah J.C. (2022). API-polymer interactions in Sepineo P600 based topical gel formulation- impact on rheology. Int. J. Pharm..

[28] Andrews G.P., Laverty T.P., Jones D.S. (2015). Rheological analysis of polymer interactions and ageing of poly(methylvinylether-co-maleic anhydride)/poly(vinyl alcohol) binary networks and their effects on mucoadhesion. J. Pharm. Sci..

[29] van Heugten A.J.P., Landman J., Petukhov A.V., Vromans H. (2018). Study of petrolatum structure: Explaining its variable rheological behavior. Int. J. Pharm..

[30] Pandey P., Ewing G.D. (2008). Rheological characterization of petrolatum using a controlled stress rheometer. Drug Dev. Ind. Pharm..

[31] Krishnaiah Y.S., Xu X., Rahman Z., Yang Y., Katragadda U., Lionberger R., Peters J.R., Uhl K., Khan M.A. (2014). Development of performance matrix for generic product equivalence of acyclovir topical creams. Int. J. Pharm..

[32] Kryscio D.R., Sathe P.M., Lionberger R., Yu L., Bell M.A., Jay M., Hilt J.Z. (2008). Spreadability measurements to assess structural equivalence (Q3) of topical formulations—A technical note. AAPS PharmSciTech.

[33] Bao Q., Burgess D.J. (2018). Perspectives on physicochemical and in vitro profiling of ophthalmic ointments. Pharm. Res..

[34] Park E.-K., Song K.-W. (2010). Rheological evaluation of petroleum jelly as a base material in ointment and cream formulations: Steady shear flow behavior. Arch. Pharmacal Res..

[35] Park E.K., Song K.W. (2011). Rheological evaluation of petroleum jelly as a base material in ointment and cream formulations: Linear viscoelastic behavior. J. Pharm. Investig..

