# Supplementary material for: Rheological Characterization of Lemon Oil-Loaded Eucerin Cream and Aquaphor Ointment
Source: Pharmaceuticals (Basel). 2025 Dec 2;18(12):1838. doi: 10.3390/ph18121838 (PMC12735537; doi:10.3390/ph18121838)
Supplement: Supplementary file 1 [file pharmaceuticals-18-01838-s001.zip › pharmaceuticals-4010902-supplementary.pdf]

Rheological Characterization of Lemon Oil-Loaded Eucerin Cream and Aquaphor Ointment

Shorouq Alzahrani[1], Jinsong Hao[1]*

[1] Department of Pharmaceutical & Clinical Sciences, College of Pharmacy & Health Sciences, Campbell University, Buies Creek, North Carolina, 27506, USA

Corresponding author:

Jinsong Hao, Ph.D.

Campbell University

PO Box 1090

Buies Creek, NC 27506

Email: jhao@campbell.edu

Supplementary Materials

Table S1. Viscosities (Pa·s) at representative flow rates (s^{-1}) for Eucerin and Aquaphor formulations containing various concentrations (% *w/w*) of lemon oil measured in flow sweep tests.

Eucerin formulations					
Shear rate (s^{-1})	Control	10%	20%	30%	40%
0.1	310.122	362.401	262.816	216.358	151.307
1	61.020	39.832	25.256	20.182	12.665
10	7.542	3.603	2.709	2.592	1.916
100	0.979	0.619	0.512	0.467	0.315
Aquaphor formulations					
Shear rate (s^{-1})	Control	5%	10%	15%	20%
0.1	637.026	904.562	241.456	161.719	107.261
11	27.945	18.149	11.943	9.130	6.577
55	5.364	3.768	2.284	1.635	1.080
100	2.278	1.772	1.166	0.858	0.582

Table S2. Slopes of the plots of tan delta versus frequency for Eucerin and Aquaphor formulations containing various concentrations (% *w/w*) of lemon oil measured in flow sweep tests. The slopes were linearly changed with lemon oil concentration.

Eucerin formulations	Control (0%)	10%	20%	30%	40%
Slope	-0.1982	-0.1341	-0.1066	-0.0623	-0.0128
Linear relationship	$y = 0.0044x - 0.1913$; $R^2 = 0.9875$				
Aquaphor formulations	Control (0%)	5%	10%	15%	20%
Slope	0.0172	0.0441	0.0656	0.0838	0.0941
Linear relationship	$y = 0.0039x + 0.0223$; $R^2 = 0.9749$				

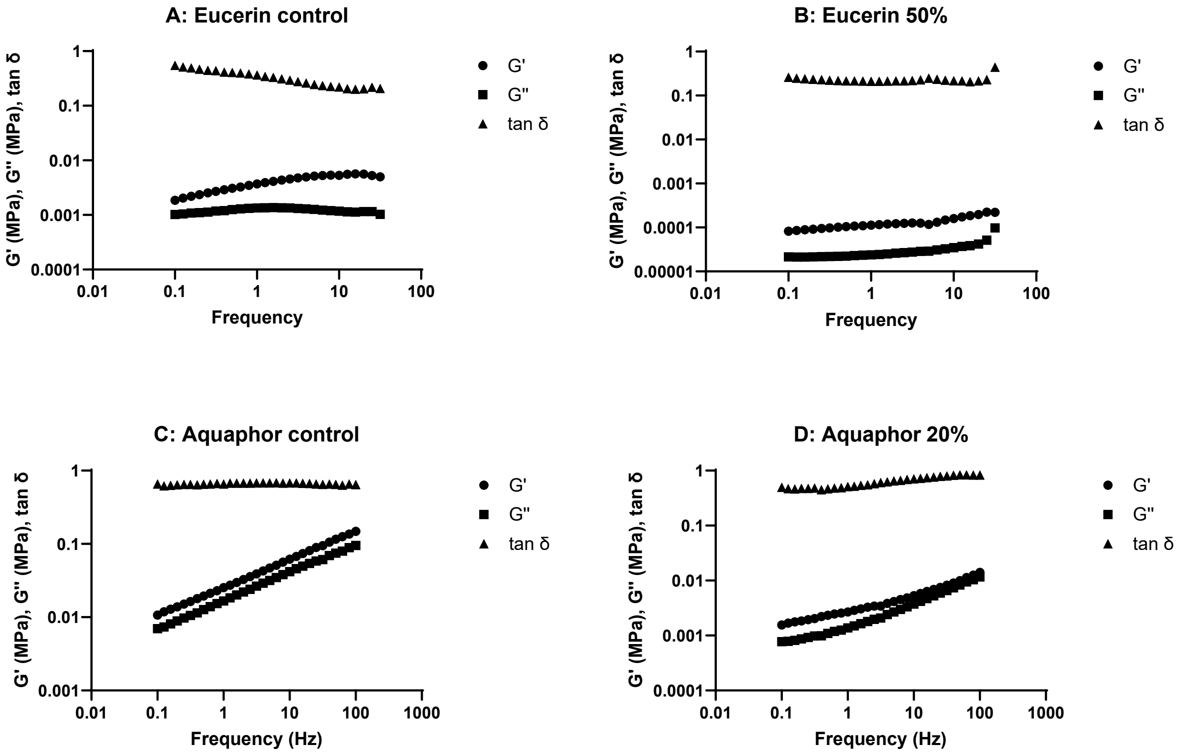

Figure S1. Plots of storage modulus (G'), loss modulus (G''), and tan delta (tan δ) over oscillatory frequency obtained in the preliminary oscillation frequency sweep studies to determine maximum loading of lemon oil for Eucerin and Aquaphor formulations.

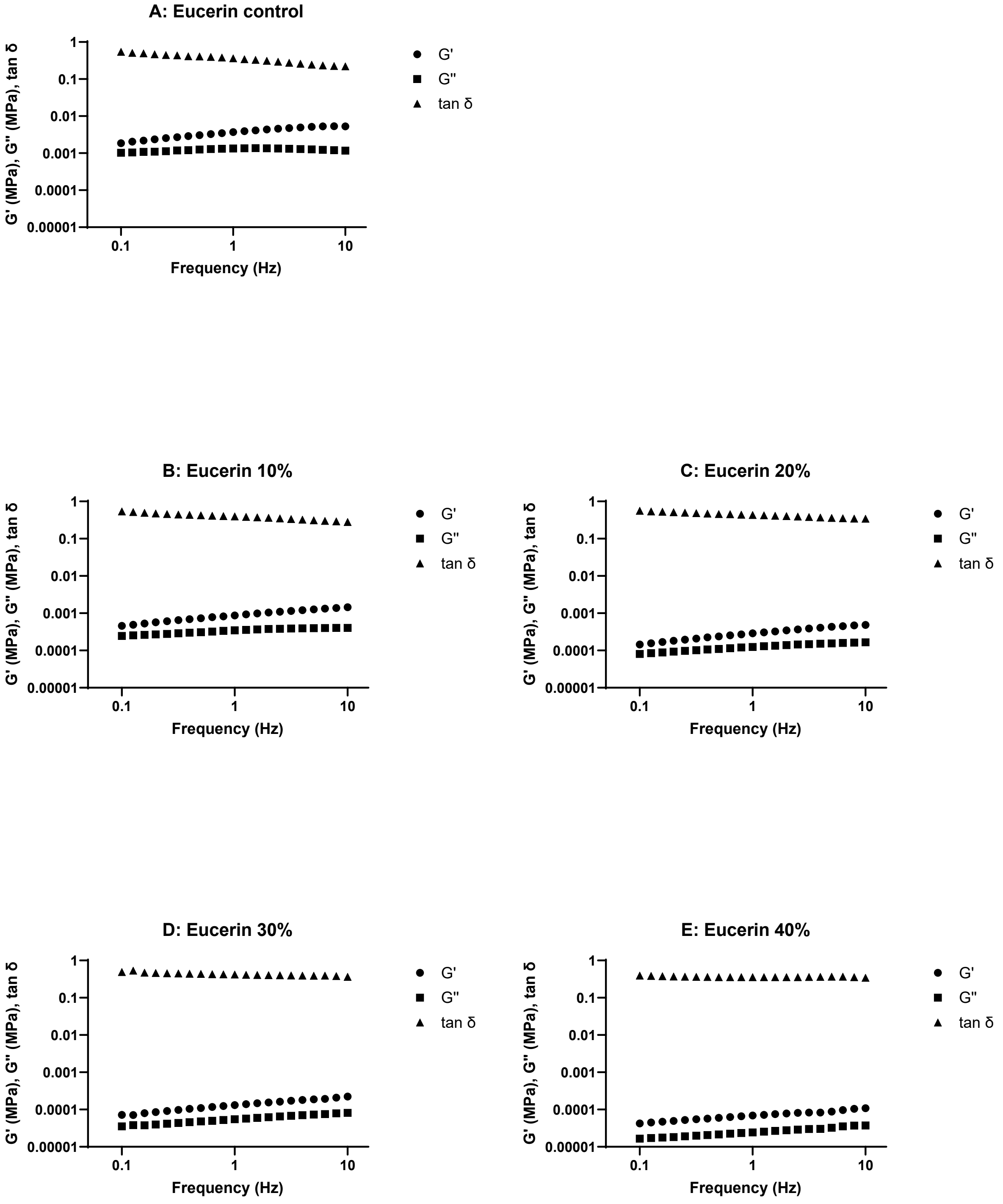

Figure S2. Plots of storage modulus (G'), loss modulus (G''), and tan delta (tan δ) over oscillatory frequency for lemon oil loaded Eucerin formulations.

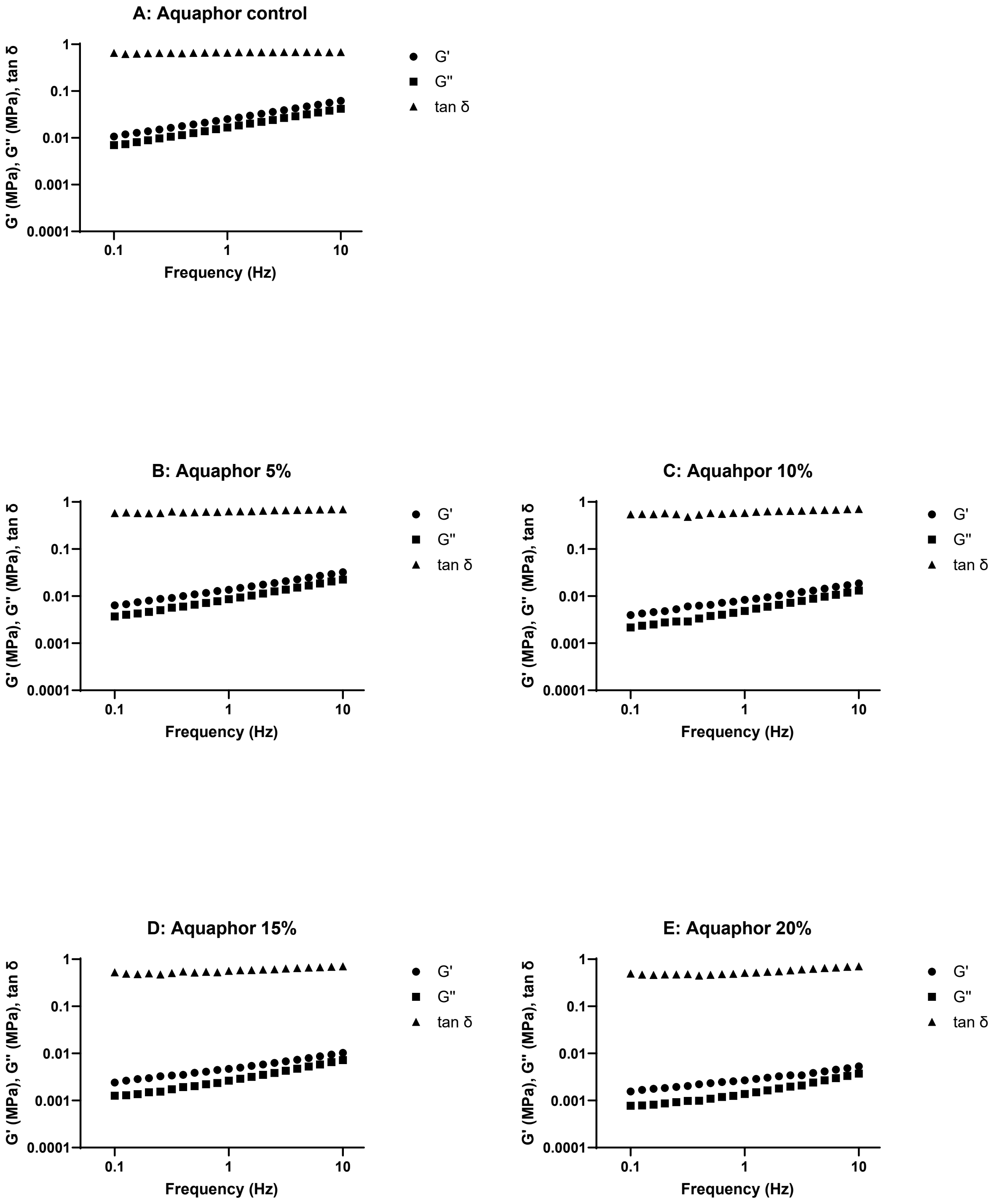

Figure S3. Plots of storage modulus (G'), loss modulus (G''), and tan delta (tan δ) over oscillatory frequency for lemon oil loaded Aquaphor formulations.

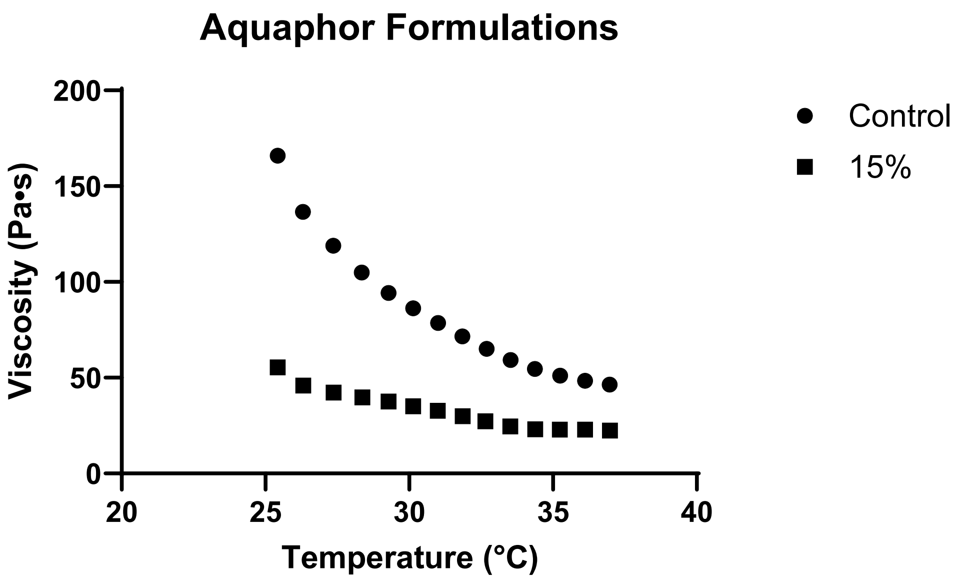

Figure S4. Viscosity profiles of Aquaphor control and Aquaphor 15% formulations as a function of testing temperature.

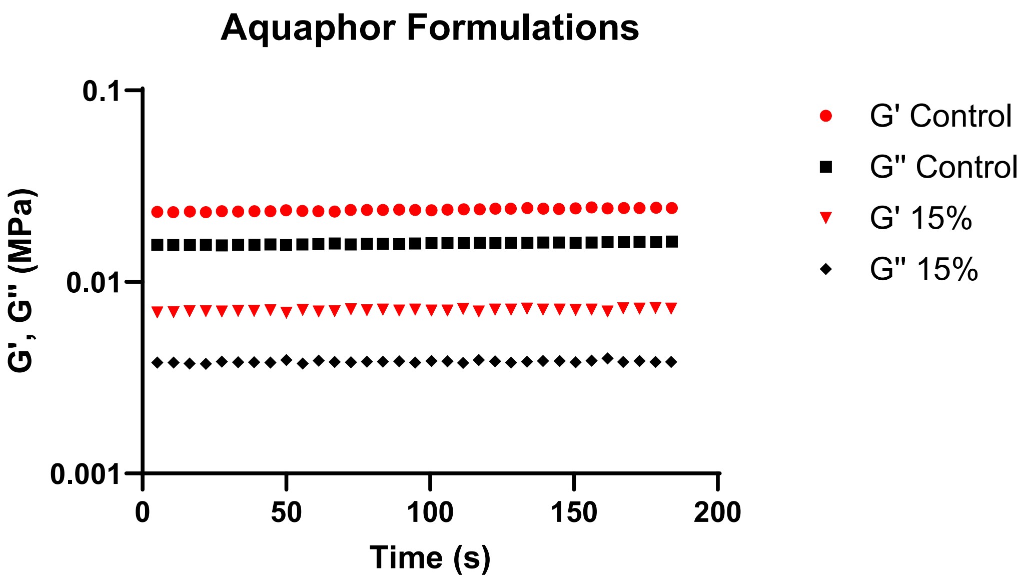

Figure S5. Plots of storage modulus (G') and loss modulus (G'') over time for Aquaphor control and Aquaphor 15% formulations.

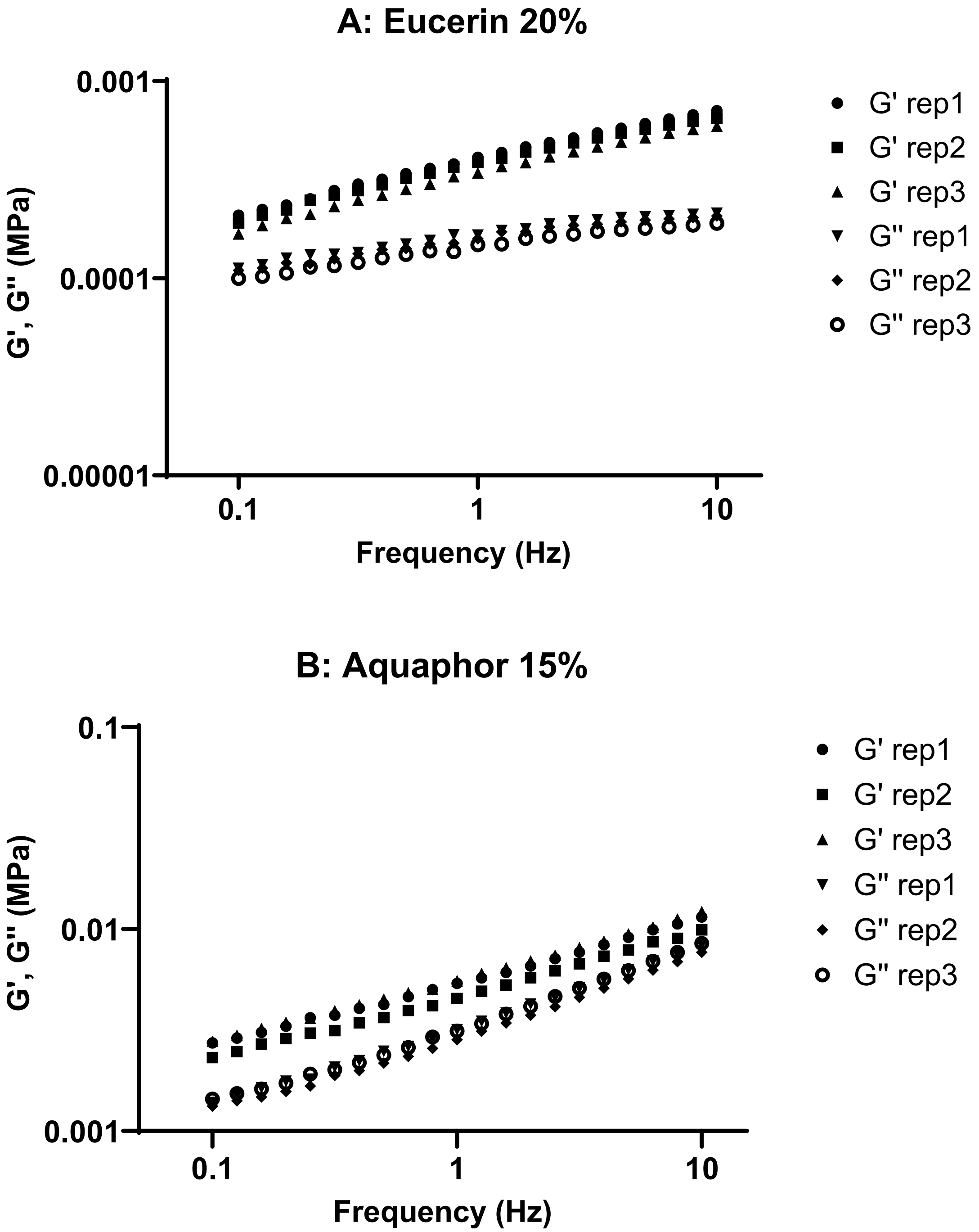

Figure S6. Plots of storage modulus (G') and loss modulus (G") over oscillatory frequency for (A) Eucerin 20% and (B) Aquaphor 15% formulations after three tests under the same testing condition.
